# Multidrug Resistance-Associated Protein 1 (MRP1) mediated vincristine resistance: effects of N-acetylcysteine and Buthionine Sulfoximine

**DOI:** 10.1186/1475-2867-5-22

**Published:** 2005-07-24

**Authors:** Ilhan Akan, Selma Akan, Hakan Akca, Burhan Savas, Tomris Ozben

**Affiliations:** 1 Akdeniz University, Faculty of Medicine, Department of Biochemistry, 07070 Antalya, Turkey; 2 Pamukkale University, Faculty of Art&Science, Department of Biology, Denizli, Turkey; 3 Akdeniz University, Faculty of Medicine, Department of Internal Medicine, Division of Oncology, 07070 Antalya, Turkey

**Keywords:** MRP1, vincristine, HEK293, N-acetylcysteine, BSO, GSH

## Abstract

**Background:**

Multidrug resistance mediated by the multidrug resistance-associated protein 1 (MRP1) decreases cellular drug accumulation. The exact mechanism of MRP1 involved multidrug resistance has not been clarified yet, though glutathione (GSH) is likely to have a role for the resistance to occur. N-acetylcysteine (NAC) is a pro-glutathione drug. DL-Buthionine (S,R)-sulfoximine (BSO) is an inhibitor of GSH synthesis. The aim of our study was to investigate the effect of NAC and BSO on MRP1-mediated vincristine resistance in Human Embryonic Kidney (HEK293) and its MRP1 transfected 293MRP cells. Human Embryonic Kidney (HEK293) cells were transfected with a plasmid encoding whole MRP1 gene. Both cells were incubated with vincristine in the presence or absence of NAC and/or BSO. The viability of both cells was determined under different incubation conditions. GSH, Glutathione S-Transferase (GST) and glutathione peroxidase (GPx) levels were measured in the cell extracts obtained from both cells incubated with different drugs.

**Results:**

N-acetylcysteine increased the resistance of both cells against vincristine and BSO decreased NAC-enhanced MRP1-mediated vincristine resistance, indicating that induction of MRP1-mediated vincristine resistance depends on GSH. Vincristine decreased cellular GSH concentration and increased GPx activity. Glutathione S-Transferase activity was decreased by NAC.

**Conclusion:**

Our results demonstrate that NAC and BSO have opposite effects in MRP1 mediated vincristine resistance and BSO seems a promising chemotherapy improving agent in MRP1 overexpressing tumor cells.

## Background

The acquisition of resistance to anticancer agents used in chemotherapy is the main cause of treatment failure in malignant disorders, provoking tumours to become resistant during treatment, although they initially respond to it [1-4]. Resistance of cancer cells to a single drug is usually accompanied by resistance to other drugs with different structures and cellular targets [3,4]. Identifying the mechanisms leading to intrinsic or acquired multidrug resistance (MDR) is important in developing more effective therapies. At least, two proteins are well-known for causing MDR. Both proteins, the *MDR1 *gene encoded-Pgp and MRP1 are members of the ATP binding cassette transporter superfamily. Despite their common involvement in MDR, there are clear differences in function and substrate specifity of Pgp and MRP1 [5]. Pgp transports neutral, or positively charged, hydrophobic compounds [5]. In contrast, MRP1 extrudes conjugated organic anions from cells and is known as multispecific aniontransporter (MOAT) [4,6,7]. The exact mechanism of MRP1 involved multidrug resistance remains unknown, although GSH is likely to have a role for the resistance to occur. Thus, clarifying the mechanism of action of MRP1 in cell lines ortumors overexpressing MRP1 and the search for inhibitors of drug transport can give new insights in future experiments and therapies.

Multidrug resistance protein (MRP1) mediated drug resistance occurs against a broad spectrum of natural product drugs like vincristine, although the mechanisms have not been exactly understood and it has not been possible to demonstrate that MRP1 can actively transport unmodified forms of vincristine [8]. Vincristine is a vinca alcaloid type drug and a widely used chemotherapeutic agent for the treatment of acute leukemia and solid tumors [9]. Efflux of hydrophobic natural product anticancer drugs agents such as vincristine from cells expressing MRP1 is thought to require GSH [10,11]. The nature of the involvement of GSH is not fully clarified, though co-transport of GSH is now believed to take place [8,10,12]. GSH is the most abundant non-protein intracellular thiol containing compound that is a key molecule in MRP1-mediated MDR [3,13]. It was shown that ATP-dependent uptake of vincristine by MRP-enriched, inside-out membrane vesicles could be stimulated by physiological concentrations of GSH [14]. It is suggested that increased MRP1 expression without an increase in GSH biosynthesis would not cause any drug resistance in tumor cells, but would result in cell death [15]. GSH conjugates with drugs catalyzed by the enzyme GST and causes their subsequent removal from the cells [15]. BSO inhibits GSH synthesis by irreversible inhibition of γ-glutamyl cysteine synthase and has no other known effect on cells [3,11,16]. N-acetylcysteine is a thiol antioxidant and cysteine source for GSH synthesis [17]. The study aimed to define the mechanism of action of vincristine and the effects of NAC and BSO on MRP1-mediated vincristine resistance in Human Embryonic Kidney (HEK293) and its MRP1 transfected 293MRP cells. For this purpose, HEK293 and 293MRP cells were incubated with vincristine in the presence or absence of NAC and/or BSO. Vincristine cytotoxicity, cell viability and the effect of vincristine on cellular GSH levels, GST and GPx enzyme activities were determined in both cell groups in the presence or absence of NAC at two different concentrations.

## Results

**Figure 1:**
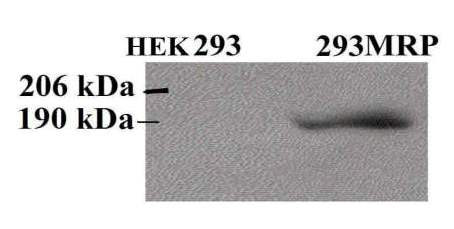
Western Blot Detection of MRP1 in Human Embryonal Kidney Cell Line Transfected with the MRP1 gene.

Western Blot analysis using monoclonal QCRL-1 anti-MRP1 antibody demonstrated MRP1 expression in 293MRP cells, unlike HEK293 cells (Fig 1).

### Cytotoxic Activity of Vincristine

**Figure 2:**
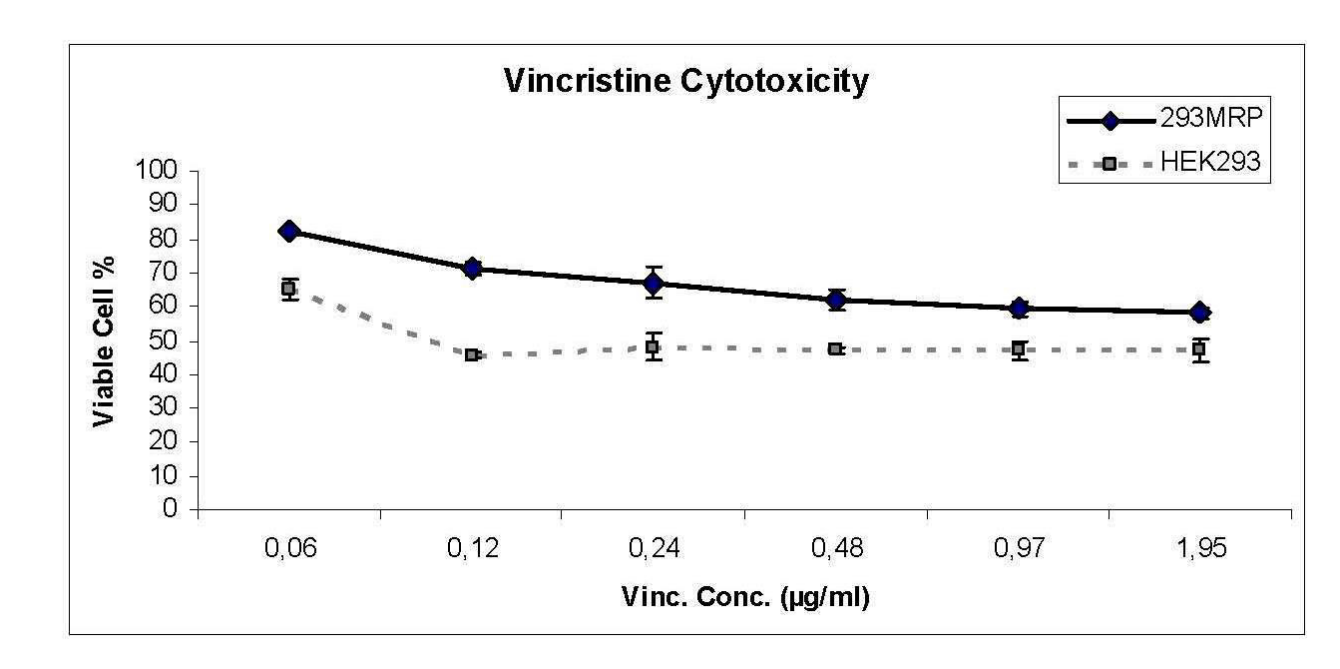
Cell viability of MRP1 and HEK293 cells against different concentrations of vincristine.

The experiments were repeated 3 times and the results obtained from these repetitions were averaged. The cytotoxic effect of different concentrations of vincristine on HEK293 cells was shown in Figure 2. The lethal concentration (LD_50_) of vincristine was found as 0.156 μg/ml on HEK293 cells using crystal violet method (Fig 2). This concentration of vincristine was applied for incubation of the cells.

### Effects of NAC on vincristine cytotoxicity

**Figure 3:**
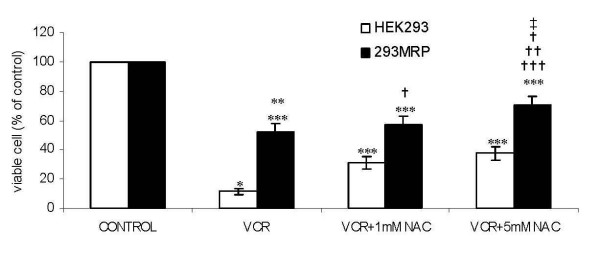
Effect of N-acetylcysteine (NAC) on vincristine cytotoxicity in human embryonic kidney (HEK293) and 293MRP Cells. * p < 0.05 vs untreated control HEK293 cells ** p < 0.05 vs untreated control 293MRP cells *** p < 0.05 vs HEK293 cells treated with Vinc † p < 0.05 vs HEK293 cells treated with Vinc + 1 mM NAC †† p < 0.05 vs HEK293 cells treated with Vinc + 5 mM NAC ††† p < 0.05 vs 293MRP cells treated with Vinc ‡ p < 0.05 vs 293MRP cells treated with Vinc + 1 mM NAC.

The viability of HEK293 and 293MRP cells treated with vincristine was significantly lower than the respective untreated control cells (11.4 ± 2.3% and 52.4 ± 5.2% respectively, p < 0.5) (Fig 3). 293MRP cells were more resistant to vincristine than HEK293 cells. The lowest level in cell viability was observed in HEK293 cells. Both cells were incubated with 1 or 5 mM NAC in the presence of vincristine. N-acetylcysteine supplementation at both concentrations enhanced significantly the resistance of 293MRP and HEK293 cells against vincristine cytotoxicity compared to their respective untreated control cells (p < 0.05). The viability of HEK293 cells increased significantly (p < 0.05) from 11.4 ± 2.3% to 31.1 ± 4.1% with 1 mM NAC and to 37.5 ± 4.7% with 5 mM NAC. The viability of 293MRP cells increased significantly (p < 0.05) from 52.4 ± 5.2% to 57.2 ± 5.4% with 1 mM NAC and to 70.1 ± 6.2% with 5 mM NAC. There was no significant difference between the viability of HEK293 cells treated with two different concentrations of NAC, but 5 mM NAC was more effective in 293MRP cells compared to the 1 mM NAC (p < 0.05).

### Effect of BSO on vincristine cytotoxicity and survival promoting action of NAC

**Figure 4:**
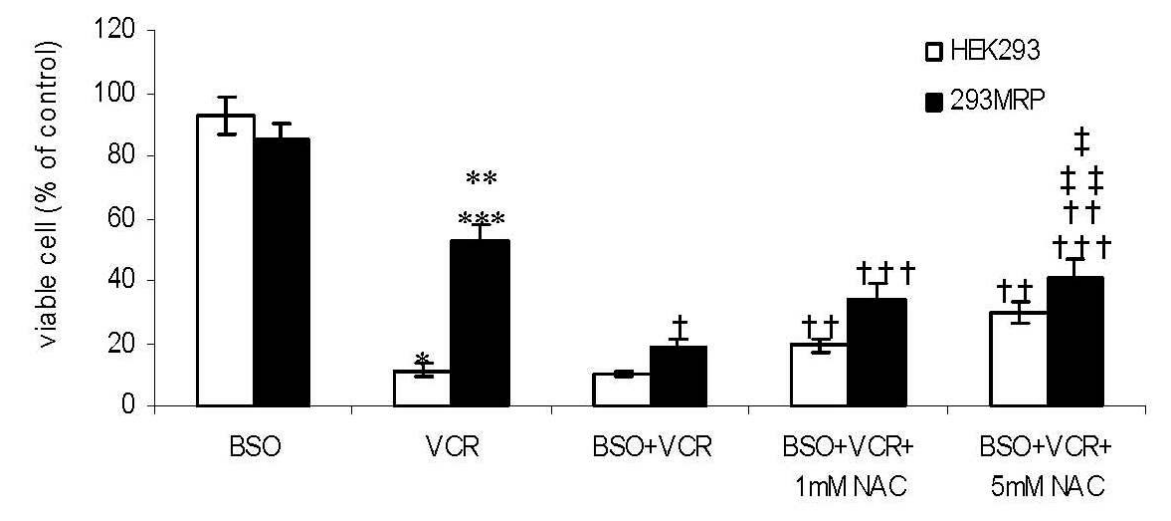
Effect of DL-Buthionine (S,R)-sulfoximine (BSO) on vincristine cytotoxicity and survival promoting effect of N-acetylcysteine (NAC) in human embryonic kidney (HEK293) and 293MRP Cells. * p < 0.05 vs HEK293 cells pretreated with BSO ** p < 0.05 vs 293MRP cells pretreated with BSO *** p < 0.05 vs HEK293 cells treated with Vinc † p < 0.05 vs 293MRP cells treated with Vinc †† p < 0.05 vs HEK293 cells treated with BSO+Vinc and HEK293 cells treated with Vinc ††† p < 0.05 vs 293MRP cells treated with BSO+Vinc ‡ p < 0.05 vs 293MRP cells treated with BSO+Vinc+1 mM NAC ‡‡ p < 0.05 vs HEK293 cells treated with BSO+Vinc+1 mM NAC.

Cells were pretreated with 100 μM BSO for 24 hour before drug treatments. The viability of 293MRP and HEK293 cells pretreated with BSO was not different significantly (85 ± 5.3% and 93 ± 6.1%, respectively. p > 0.05) (Fig 4). After inhibition of GSH synthesis with BSO, 293MRP cells lost their vincristine resistance significantly from 52.4 ± 5.2% to 19.0 ± 1.9% (p < 0.05) (Fig 4). Pretreatment with BSO didnot affect the viability of HEK293 cells treated with vincristine (11.4 ± 2.3% vs 10.2 ± 1.2%). N-acetylcysteine at both concentrations increased significantly the viability of 293 MRP cells pretreated with BSO against vincristine (from 19.0 ± 1.9% to 33.6 ± 5.4 with 1 mM NAC and to 40.5 ± 6.2% with 5 mM NAC). Similar increase was observed in HEK293 cells under the same conditions (from 10.2 ± 1.2% to 19.2 ± 2.4 with 1 mM NAC and to 29.9 ± 3.2% with 5 mM NAC). Pretreatment with BSO antagonized partly the increases in the viability of both cells caused by treatment with NAC compared to the increases caused by NAC alone (Fig 3 and Fig 4). In other words, NAC increased less the viability of both cells pretreated with BSO than the cells treated with only NAC against vincristine.

### Effect of vincristine and NAC on cellular GSH concentrations

**Figure 5:**
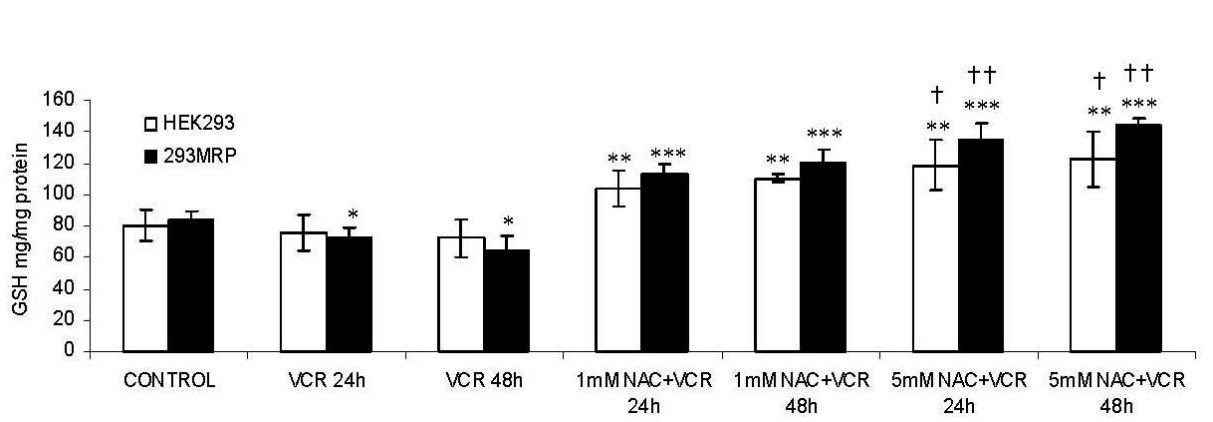
Effects of Vincristine and N-acetylcysteine (NAC) on intracellular glutathione (GSH) levels in human embryonic kidney (HEK293) and 293MRP Cells. * p < 0.05 vs untreated control 293MRP cells ** p < 0.05 vs untreated control HEK293 cells and HEK293 cells treated with Vinc for 24 and 48 hour *** p < 0.05 vs untreated control 293MRP cells and 293MRP cells treated with Vinc for 24 and 48 hour † p < 0.05 vs HEK293 cells treated with Vinc+1 mM NAC for 24 and 48 hour †† p < 0.05 vs 293MRP cells treated with Vinc+1 mM NAC for 24 and 48 hour.

Cellular GSH levels were measured after 24 and 48 hour vincristine treatments in the presence or absence of NAC at two different concentrations. Cellular GSH concentrations were not different in untreated control HEK293 and 293MRP cells (80.2 ± 4.6 μg mg^-1 ^protein and 84.6 ± 4.9 μg mg^-1 ^protein, respectively) (Fig 5). Glutathione levels decreased not significantly after 24- and 48-h vincristine treatments in HEK293 cells from 80.2 ± 4.6 μg mg^-1 ^protein to 75.9 ± 3.8 μg mg^-1 ^protein and to 72.2 ± 3.5 μg mg^-1 ^protein (p > 0.05) and in 293MRP cells from 86 ± 4.9 μg mg^-1 ^protein to 72.4 ± 3.4 μg mg^-1 ^protein and to 64.9 ± 3.2 μg mg^-1 ^protein (p < 0.05). N-acetylcysteine at both concentrations caused a significant increase in GSH concentrations in both cells treated with vincristine for both incubation times, in comparison to untreated control cell lines and cells treated with only vincristine (p < 0.05) (Fig 5).

### Effect of vincristine and NAC on the activity of cellular Glutathione S-Transferase and Glutathione Peroxidase

**Figure 6:**
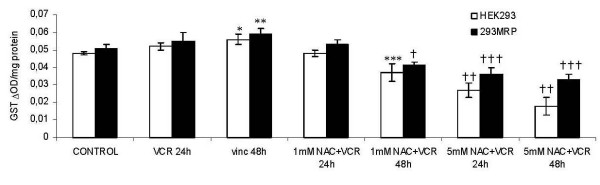
Effects of Vincristine and N-acetylcysteine (NAC) on Glutathione S-transferase Activity (GST) in human embryonic kidney (HEK293) and 293MRP Cells. * p < 0.05 vs untreated control HEK293 cells ** p < 0.05 vs untreated control 293MRP cells *** p < 0.05 vs untreated control HEK293 cells and HEK293 cells treated with Vinc for 24 and 48 hour † p < 0.05 vs untreated control 293MRP cells and 293MRP cells treated with Vinc for 24 and 48 hour †† p < 0.05 vs untreated control HEK293 cells and HEK293 cells treated with Vinc and HEK293 cells treated with Vinc+1 mM NAC for 24 and 48 hour ††† p < 0.05 vs untreated control 293MRP cells and 293MRP cells treated with Vinc and 293MRP cells treated with Vinc+1 mM NAC for 24 and 48 hour.

Glutathione S-Transferase activity in both untreated control cell lines was not significantly different. 48 hour, but not 24 hour incubation with vincristine significantly increased the GST activity in both cell lines comparing to the corresponding untreated control cells (Fig 6). N-acetylcysteine (1 mM) for 48 hour caused a significant decrease in GST activity in 293MRP and HEK293 cells compared with nontreated control cells and cells treated with only vincristine for both incubation times. 5 mM NAC at both incubation times caused a significant decrease in the GST activity in both cell lines compared to nontreated control cells, cells treated with vincristine only and cells treated with vincristine + NAC (1 mM) for both incubation times (Fig 6).

**Figure 7:**
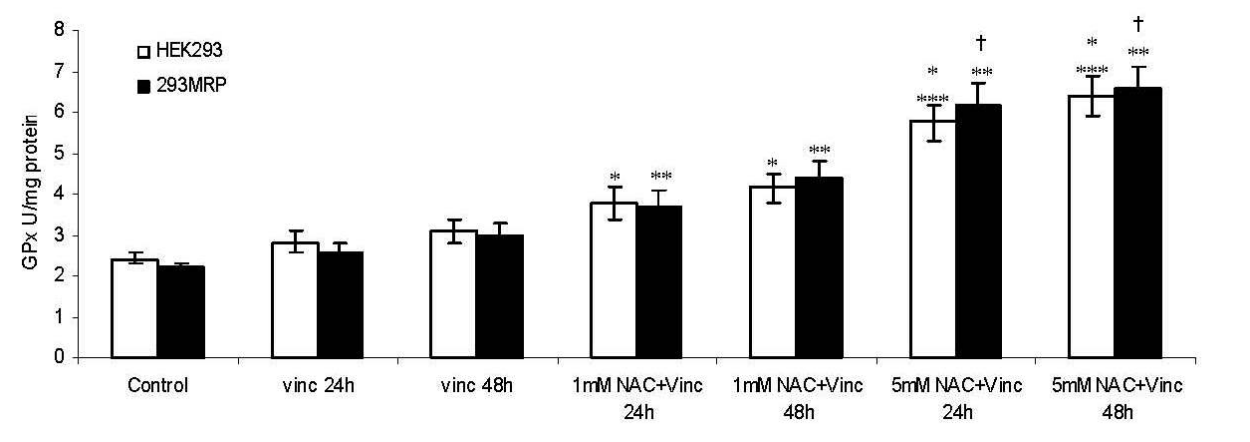
Effects of Vincristineand N-acetylcysteine (NAC) on Glutathione Peroxidase (GPx) Activity in human embryonic kidney (HEK293) and 293MRP Cells. * p < 0.05 vs untreated control HEK293 cells ** p < 0.05 vs untreated control 293MRP cells *** p < 0.05 vs untreated control HEK293 cells and HEK293 cells treated with Vinc and HEK293 cells treated with Vinc+1 mM NAC for 24 and 48 hour † p < 0.05 vs untreated control 293MRP cells and 293MRP cells treated with Vinc and 293MRP cells treated with Vinc+1 mM NAC for 24 and 48 hour.

There was no significant difference in GPx activity between HEK293 and 293MRP cells (2.4 ± 0.2 IU mg^-1 ^protein and 2.2 ± 0.1 IU mg^-1 ^protein, respectively) (Fig 7). Non-significant increases in GPx activity were observed after vincristine treatment for both incubation times in HEK293 (2.8 ± 0.3 IU mg^-1 ^protein for 24 h vincristine incubation and 3.1 ± 0.3 IU mg^-1 ^protein for 48 h vincristine incubation) and 293MRP cells (2.6 ± 0.2 IU mg^-1 ^protein for 24 h vincristine incubation and 3.0 ± 0.3 IU mg^-1 ^protein for 48 h vincristine incubation) compared to untreated control cells (p > 0.05). N-acetylcysteine (1 mM) incubation for 24 and 48 hours increased GPx activity in both cell lines compared to untreated control cells. 5 mM NAC incubation for 24 and 48 hours increased the GPx activity significantly in HEK293 and 293MRP cells compared to the other cell groups (p < 0.05) (Fig 7).

## Discussion

In our experiments, the viability of MRP1 transfected cells (293MRP) treated with vincristine was higher than human embryonic kidney (HEK293). Our results are in accordance with O'Brien and co-workers who reported that MRP1 confers resistance to doxorubicin, etoposide, and vincristine in NIH 3T3 fibroblast cell line [18]. Our experiments with N-acetylcysteine (NAC) and DL-Buthionine (S,R)-sulfoximine (BSO) showed that MRP1 mediated vincristine resistance largely depends on GSH and this is in accordance with previous data [3,18,19]. N-acetylcysteine supplementation at both concentrations increased the survival rate of vincristine treated HEK293 and 293MRP cells which had increased GSH levels confirming that the viability depends on the level of GSH (Fig 3 and Fig 4). After inhibition of GSH synthesis with BSO, 293MRP cells lost their vincristine resistance (Fig 4). Similar results were described previously in different cell lines overexpressing MRP1 [3,16].

We compared the viability of both cells treated with vincristine and NAC in the presence and absence of BSO. N-acetylcysteine at both concentrations increased significantly the viability of 293MRP and HEK293 cells pretreated with BSO against vincristine, but these increases were lower in comparison to the corresponding cells untreated with BSO (Fig 3 and Fig 4). Pretreatment with BSO antagonized partly the increases in the viability of both cells caused by treatment with NAC compared to the increases caused by NAC alone. In other words, NAC increased less the viability of both cells pretreated with BSO than the cells treated with only NAC against vincristine. This might be explained that BSO counterbalances the effect of NAC as a precursor of GSH. This is another proof that survival promoting action of NAC depends on GSH synthesis and is in accordance with our previous findings with doxorubicin [1].

In our experiments, cellular GSH concentration decreased after vincristine treatment which might be due to GSH efflux (Fig 5). Enhanced GSH efflux has been reported in MRP1 expressing cells and this enhanced efflux can be inhibited by indomethacin and probenecid [10]. They suggested that changes in the concentrations of GSH and its oxidised form GS-SG inside cells may each influence MRP1-mediated anion transport. Furthermore, hypoxia or oxidative stress may cause depletion of glutathione (GSH). Increased oxidative stress has been reported to associate tumorigenesis [20,21] and this may play a role in GSH depletion [22,23]. which in turn may affect efflux of drugs. The higher GPx activity in vincristine treated cells might be a compensatory effect of cells against depletion of GSH (Fig 6).

It has been hypothesized that vincristine resistance of myeloblasts is related to its degradation by myeloperoxidase (MPO) [9]. Myeloperoxidase (MPO) catalyzes the formation of HOCl from H_2_O_2 _and chloride ion. It was shown oxidation by HOCl is the final step in vincristine degradation in both a cell free system and in cultures leukemic cell lines. Oxidation of anti-neoplastic drugs may cause a reduction in efficacy or an increase in toxicity. This could lead to a decrease in the therapeutic index. Inhibition of MPO in these different disease states could eliminate this intra- and extracellular oxidation pathway and could effectively increase the therapeutic index.

The identification of MRP1 as an important glutathione-conjugate efflux pump raises the possibility that MRP1 and GST may act in synergy to confer cellular resistance to some of these compounds [3,14,24,25]. It is not clear yet if glutathione is either co-transported as a GS-conjugate with vincristine or activates MRP1 for vincristine transport [4]. Studies so far showed conjugation with GSH and extrusion are not the major pathway [4]. Co-expression with MRP1 of any of the human GST isozymes A1-1, M1-1, or P1-1 failed to augment MRP1-associated resistance to drugs including doxorubicin, vincristine, etoposide, and mitoxantrone [4,24]. This might be an evidence that vincristine is not conjugated with GSH, but co-transported with GSH in MRP1 mediated drug resistance.

In our study, NAC supplementation decreased GST activity level in both cell lines (Fig 7). This might be explained that NAC may spontaneously form conjugates with vincristine, therefore decreasing the need for GST activity for conjugation. Although, it is not clear whether NAC spontaneously conjugates with vincristine, it is known that mercapturic acids (N-acetylcysteine S-conjugates) are spontaneously formed, released into the circulation and delivered to the kidney for excretion in urine [26-28]. Similarly, Weigand et al reported attenuation of GST activity after NAC supplementation [29].

## Conclusion

Our results demonstrate that NAC enhances MRP1-mediated vincristine resistance and this effect depends on GSH synthesis. DL-Buthionine (S,R)-sulfoximine seems a promising chemotherapy improving agent in MRP1 overexpressing tumor cells. This finding might be relevant and have an implication in cancer patients undergoing chemotherapy.

## Methods

### Materials

Dulbeccos' Modified Eagles' Medium (DMEM), NAC, BSO, geneticin, Feotal Bovine Serum (FBS), and other chemicals were purchased from Sigma-Aldrich Corp. St. Louis, MO, USA. Vincristine was obtained from Oncology Department of Akdeniz University Hospital. The plasmid (pcDNA3.1/MRPK) encoding the whole MRP1 gene was kindly provided by Dr. Susan Cole from Oueen's University, Ontario Canada. Protein Assay Kit was purchased from Bio-Rad Laboratories Ltd., Herts, UK. Monoclonal anti-MRP1 QCRL-1 antibody was obtained from Centocor Inc., Malvern, PA, USA. Horseradish peroxidase (HRP) conjugated seconder goat-anti mouse antibody was purchased from Santa-Cruz Biotechnology Inc., Santa Cruz, CA, USA.

### Cell lines

Human embryonic kidney cell line, HEK293, was grown in DMEM, supplemented with 10% heat inactivated FBS, 2 mM L-glutamin, and 1% antibiotic-antimycotic solution. Cell cultures were kept at 37°C in a humid atmosphere containing 5% CO_2_.

### Transfection

Cells (1 × 10^6 ^cells in 100 mm dish) were transfected with the plasmid (pcDNA3.1/MRPK) encoding the whole MRP1 gene. The transfection was made according to the calcium phosphate transfection method [30]. Sixteen hours after the transfection, the cells were feeded with DMEM supplemented with 400 μg/ml geneticin.

### Preparation of Membrane Enriched Fractions and Immunoblotting

For immunoblotting of the 293MRP and HEK293 cells, membrane enriched fractions were prepared according to Grant et al [31]. Briefly, cell pellet was resuspended in the collection buffer (10 mM Tris-HCL, pH 7.4, 10 mM KCl, 1.5 mM MgCl_2 _and protease inhibitors), homogenized on ice in a Potter-Elvejhem tissue homogenizer. The intact cells and nuclei were removed by centrifugation at 800 g at +4°C, and the supernatant was further centrifuged at 100 000 g at +4°C for 20 minutes to prepare the membrane enriched fractions. The pellet was resuspended in buffer (10 mM Tris-HCl pH 7.4, 125 mM sucrose, and protease inhibitors). The protein suspension was mixed with solubilizing buffer (4 M urea, 0.5% Sodiumdodecylsulphate (SDS), and 50 mM dithiotreitol) and equal amounts of proteins were subjected to SDS-PAGE (SDS-Polyacrylamide gel electrophoresis) on 7% polyacrylamide gels, then transferred onto nitrocellulose sheet for overnight at 40 volt, and analysed by immunoblotting with anti-MRP1 monoclonal QCRL-1 antibody.

### Cell Viability Assays

Cell viability was assayed using the crystal violet method [32]. 3 × 10^4 ^cells were seeded in a 96 well microplate. After 24 hours, cells were incubated with 0.06–1000 μg/ml vincristine for 72 hours. LD_50 _for vincristine was determined to be 0.156 μg/ml and this dose of vincristine was used in the rest of the experiments. Both HEK293 and 293MRP cells were incubated with vincristine (0.156 μg/ml) in the presence or absence of NAC (1 and 5 mM) for 72 hours at 37°C in a humid atmosphere containing 5% CO_2_. At the end of incubation period, the medium was replaced by 0.5% crystal-violet (w/v; in 50% methanol) solution. Plates were incubated for 10 min at room temperature, washed with water and adsorbed dye was eluted out with Na-citrate (0.1 M Na-citrate in 50% ethanol, pH 4.2). Absorbance, which was proportional to cell viability, was measured at a wavelength of 600 nm. Cell viability was monitored as the percentage of viable cells comparing to control, untreated cells. For BSO experiments, cells were pretreated with 100 μM BSO for 24 hours before incubating them with vincristine with or without NAC for 72 hours as described above.

### Preparation of Cell Extract for GSH and Enzyme Measurements

Cell extracts were prepared as described by Bravard et al with a slight modification [33]. 10^6 ^cells were seeded in a cell culture dish and incubated for 24 hours. After incubation with vincristine in the presence or absence of NAC for 24 or 48 hours, medium was discharged and cells were washed with phosphate buffered saline (PBS), collected in potassium phosphate buffer (50 mM, pH 7.4) with cell scraper and repeatedly freezed and thawed in liquid nitrogen for four times, and then centrifuged at 10 000 g for 10 minutes at 4°C. The supernatant was used for enzyme activities and GSH measurements. Protein concentrations were determined using Bio-Rad protein assay kit. All measurements were adjusted by dividing with the protein content of each sample.

### Reduced Glutathione Assay

Cellular GSH concentrations were determined as described by Virgil et al [34]. Briefly, the supernatant was deproteinized and GSH content was monitored spectrophotometrically with 5-5' dithiobis(2-nitrobenzoic acid) (DTNB) at a wavelength of 412 nm. The GSH concentration was evaluated using a standart curve of known amounts of GSH. Results are expressed as μg/mg protein.

### Glutathione S-Transferase Activity Assay

Glutathione S-Transferase (GST) activity was measured at 340 nm wavelength in the presence of 1-cloro-2,4-dinitrobenzene (CDNB), GSH and sodium phosphate buffer (pH 6.5) at 30°C for 6 minutes [33,35]. Results are expressed as ΔOD / mg protein.

### Glutathione Peroxidase Activity Assay

Glutathione Peroxidase (GPx) activity was determined using a modification of the method of Paglia and Valentine [36]. In a cuvette kept at 37°C, GPx activity was monitored at 340 nm by the absorbance of nicotinamide adenine dinucleotide phosphate (NADPH) for 3 minutes in the presence of glutathione reductase (0.5 IU), EDTA (0.3 mM) and t-buthyl hydroperoxide (0.4 mM). Results are expressed as IU/mg protein.

### Statistical Analysis

Statistical analysis was performed using Anova test with SPSS packed program for Windows version 10.0 (SPSS Inc., Chicago, IL, USA). All the experiments were repeated three times. Mean values and standard deviations (mean ± S.D.) were calculated for every variable in each cell group and were compared between the groups. p < 0.05 was selected as statistically significant.

## Competing interests

The author(s) declare that they have no competing interests.

## Authors' contributions

IA, SA and HA carried out the cell culture studies, transfection, immunoblotting, viability assays, GSH and enzyme measurements in the cell extracts. BS and TO participated in the design of the study and performed the statistical analysis, coordination and helped to draft the manuscript. All authors read and approved the final manuscript.
